# Integrated Genomic Analysis Implicates Haploinsufficiency of Multiple Chromosome 5q31.2 Genes in *De Novo* Myelodysplastic Syndromes Pathogenesis

**DOI:** 10.1371/journal.pone.0004583

**Published:** 2009-02-25

**Authors:** Timothy A. Graubert, Michelle A. Payton, Jin Shao, Richard A. Walgren, Ryan S. Monahan, John L. Frater, Mark A. Walshauser, Mike G. Martin, Yumi Kasai, Matthew J. Walter

**Affiliations:** 1 Department of Medicine, Division of Oncology, Washington University School of Medicine, St. Louis, Missouri, United States of America; 2 Siteman Cancer Center, Washington University School of Medicine, St. Louis, Missouri, United States of America; 3 Department of Pathology and Immunology, Washington University School of Medicine, St. Louis, Missouri, United States of America; 4 Genome Sequencing Center, Washington University School of Medicine, St. Louis, Missouri, United States of America; 5 Department of Genetics & Genomic Sciences, Mount Sinai School of Medicine, New York, New York, United States of America; Stanford University, United States of America

## Abstract

Deletions spanning chromosome 5q31.2 are among the most common recurring cytogenetic abnormalities detectable in myelodysplastic syndromes (MDS). Prior genomic studies have suggested that haploinsufficiency of multiple 5q31.2 genes may contribute to MDS pathogenesis. However, this hypothesis has never been formally tested. Therefore, we designed this study to systematically and comprehensively evaluate all 28 chromosome 5q31.2 genes and directly test whether haploinsufficiency of a single 5q31.2 gene may result from a heterozygous nucleotide mutation or microdeletion. We selected paired tumor (bone marrow) and germline (skin) DNA samples from 46 *de novo* MDS patients (37 without a cytogenetic 5q31.2 deletion) and performed total exonic gene resequencing (479 amplicons) and array comparative genomic hybridization (CGH). We found no somatic nucleotide changes in the 46 MDS samples, and no cytogenetically silent 5q31.2 deletions in 20/20 samples analyzed by array CGH. Twelve novel single nucleotide polymorphisms were discovered. The mRNA levels of 7 genes in the commonly deleted interval were reduced by 50% in CD34+ cells from del(5q) MDS samples, and no gene showed complete loss of expression. Taken together, these data show that small deletions and/or point mutations in individual 5q31.2 genes are not common events in MDS, and implicate haploinsufficiency of multiple genes as the relevant genetic consequence of this common deletion.

## Introduction

**Figure 1:**
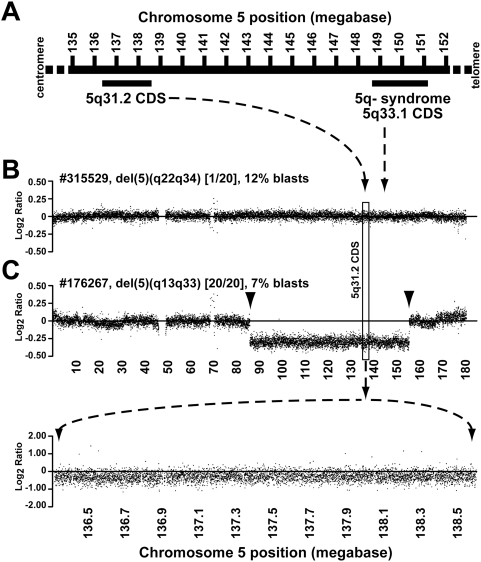
Chromosome 5q31.2 commonly deleted segment and array CGH plots. A. Diagram of the chromosome 5q31.2 commonly deleted segment (CDS) (136.3–138.6 megabases) and the 5q33.1 CDS associated with the 5q minus syndrome (148.6–151.1 megabases). The centromere is located at the left and the telomere to the right. B. Whole chromosome 5 log_2_ ratio (test/reference) plot for sample #315529 which contains del(5q) in 1/20 bone marrow cell metaphases, and has a blast count of 12%. No deletion was identified in this sample. To allow visualization of the data, each data point on the plot represents the average log_2_ ratio for consecutive probes in a 20,000 base pair region (median number of probes per bin = 46). The dashed lines indicate the 5q31.2 and 5q33.1 CDS locations depicted in panel A. C. (Upper panel) Whole chromosome 5 log_2_ ratio (test/reference) plot for sample #176267. An interstitial deletion on chromosome 5 produces a negative log_2_ ratio for probes located within the deletion. The deletion boundaries are indicated by the arrowheads. The deletion spans from base 86,152,517–155,672,191 (determined using segMNT). The log_2_ ratio deviation for the segment is consistent with the deletion of one allele. Sample #176267 contains del(5q) in 20/20 bone marrow cell metaphases (100%), and has a blast count of 7%. (Lower panel) Chromosome 5 log_2_ ratio (test/reference) plot for sample #176267 from 136.3–138.6 Mb, corresponding to the 5q31.2 CDS. No biallelic deletion was identified in this region.

Heterozygous deletions spanning two distinct regions on the long arm of chromosome 5 are among the most common abnormalities detectable by conventional cytogenetic analysis in *de novo* (6–20% of patients) and therapy-related myelodysplastic syndromes (MDS) (40% of patients) [1]. One region spans chromosome 5q33.1 (148.6–151.1 Mb) and is associated with the 5q minus syndrome, while the other spans chromosome 5q31.2 (136.3–138.6 Mb) and is associated with both *de novo* MDS and acute myeloid leukemia (AML) that progresses from MDS (**Figure 1A**) [2], [3], [4], [5], [6]. The chromosome 5q31.2 region contains 23 annotated genes, 1 pseudogene, 3 small RNAs, and 1 predicted genes (*SPOCK1, KLHL3, HNRPA0, NPY6R, MYOT, PKD2L2, C5orf5, WNT8A, NME5, BRD8, KIF20A, CDC23, GFRA3, CDC25C, FAM53C, JMJD1B, REEP2, EGR1, ETF1, HSPA9, SNORD63, LOC391836, LOC729429, CTNNA1, LRRTM2, SIL1, MATR3, SNORA74A*) [5], [6].

Prior studies have suggested that haploinsufficiency of multiple genes is the disease mechanism associated with deletions spanning 5q31.2. However, these studies were primarily designed to identify biallelic gene mutations by analyzing the residual non-deleted 5q31.2 allele using MDS samples, AML samples, or cell lines containing a cytogenetically visible 5q31.2 deletion, and they only examined 20 of the 28 5q31.2 genes [6], [7], [8], [9], [10]. Therefore, these studies did not adequately address the possibility that haploinsufficiency of a single 5q31.2 gene could arise from a heterozygous point mutation, or small microdeletion, in a 5q31.2 gene in MDS samples lacking a cytogenetic del(5q). Finding a heterozygous nucleotide mutation or microdeletion in a single 5q31.2 gene in MDS patients without evidence of del(5q) would expedite our understanding of the pathogenesis conferred by large deletions.

To address the possibility that haploinsufficiency of a single 5q31.2 gene may occur in MDS, we performed a comprehensive and systematic screen using total exonic resequencing and array comparative genomic hybridization to evaluate all 28 genes in the 5q31.2 interval from 46 MDS patients (37 lacking a 5q31.2 deletion). Our data indicate that loss of one copy of chromosome 5q31.2 genes in *de novo* MDS occurs predominantly through large cytogenetic deletions involving many genes, not a single gene, and suggest that haploinsufficiency of multiple chromosome 5q31.2 genes is likely to be the relevant genetic consequence of this common deletion.

## Materials and Methods

### Human subjects

Forty-six adult patients (age >18 years) with *de novo* MDS (no prior malignancy, antecedent chemo- or radiotherapy) were enrolled in a study at the Siteman Cancer Center at Washington University to identify genetic factors contributing to MDS initiation and progression. Approval was obtained from the Washington University institutional review board for these studies. After obtaining written informed consent, a bone marrow sample was obtained for analysis of tumor cells, and a 6-mm punch biopsy of skin was obtained for analysis of unaffected somatic cells. Bone marrow DNA was prepared using the QIAamp DNA mini kit (Qiagen, Valencia, CA) and skin DNA was prepared using a standard phenol/chloroform extraction followed by an ethanol precipitation protocol.

### Array Comparative Genomic Hybridization

Array comparative genomic hybridization (CGH) was performed using paired tumor (bone marrow) and germline (skin) genomic DNA samples (non-whole genome amplified DNA) from 20 of the 46 MDS samples that we resequenced (5/20 samples contain deletions spanning 5q31.2). These 20 samples were chosen because of sample abundance. We used a custom array CGH platform (Roche NimbleGen Systems, Inc.) containing 385,297 long oligomer (average length 51 nucleotides) probes spanning human chromosome 5 (median probe spacing ∼500 base pairs). Labeling, hybridization, washing, and scanning were performed as previously described [11]. The log_2_(test/reference) signals were analyzed using a circular binary segmentation algorithm (segMNT) [12] and a hidden Markov model (wuHMM) [13] to identify somatically acquired segmental DNA copy number changes. To call a copy number change, both algorithms required a segment to span a minimum of 5 consecutive probes. Full access to the primary array data and MIAME description are available at http://bioinformatics.wustl.edu.

### Sequencing and Pyrosequencing

Sequencing and Pyrosequencing methods have been previously described [14], [15].

### Expression profiling

CD34+ cells were purified using the autoMACS system (Miltenyi). Total RNA was prepared using Trizol (Invitrogen, Carlsbad, CA), processed, and hybridized to the human Affymetrix U133plus2 array by the Siteman Cancer Center Microarray Core Facility at Washington University according to the manufacturer's instructions (see http://pathbox.wustl.edu/~mgacore/genechip.htm for a complete list of protocols). In order to perform interarray comparisons, the data for each array was scaled to a target intensity of 1,500 using Affymetrix Microarray Suite software.

### Quantitative RT-PCR

Quantitative RT-PCR was performed using total RNA extracted from CD34+ cells and the one-step Assays-on-Demand kit (Applied Biosystems). All samples were run in duplicate. Individual cDNA samples were normalized according to their levels of beta actin transcript. The comparative Ct method was used for analysis.

### Statistical analysis

Differences in allele frequency were evaluated using Fisher's Exact test. Genotype associations were further evaluated for significance according to codominant, dominant, and overdominant genetic models by Chi-square testing. Results were corrected for multiple comparisons by the Bonferroni method. Gene expression profiling results were compared using a two-tailed t-test.

## Results

### Patient characteristics

A total of 46 patients with *de novo* MDS samples were chosen for study based on availability of high quality, abundant, paired bone marrow (tumor) and skin (germline) DNA samples (**Table 1**). Paired samples allowed us to distinguish somatic mutations from germline polymorphisms. Thirty-seven of the 46 patients did not have a chromosome 5q31.2 deletion by cytogenetics. Seven of the 9 patients with monosomy 5 or del(5q) had complex cytogenetics, and none of the 37 non-del(5q) patients had clinical evidence of 5q minus syndrome (**Table S1**). Cases were classified in accordance with the French-American-British (FAB) system upon diagnosis and banking of their bone marrow specimens. The patients include refractory anemia (RA), RA with ringed sideroblasts (RARS), RA with excess blasts (RAEB), RAEB in transformation (RAEB-T), and chronic myelomonocytic leukemia (CMML), with a median International Prognostic Scoring System (IPSS) score of 1 (range 0–3), and a median blast count of 4.5% (range 0–26%). The 46 bone marrow samples were independently reviewed by a hematopathologist (JLF) and 6 cases were reclassified as acute myeloid leukemia in the World Health Organization system (**Table S1)**. The 20 MDS samples analyzed using array CGH were chosen from the 46 MDS samples used for the resequencing studies, and had a median IPSS score of 2 (range 0–3), and a median blast count of 10% (range 1–26%) (**Table 1** **and Table S1**).

**Table 1:** Clinical characteristics of the 46 MDS patients

Characteristic	Resequencing (n = 46)	aCGH (n = 20)
**Age-yr**		
** Median**	63.5	62.5
** Range**	35–83	43–77
**Sex-no.(%)**		
** Male**	30(65)	14(70)
** Female**	16(35)	6(30)
**FAB class-no.(%)**		
** Refractory anemia**	22(48)	2(10)
** Refractory anemia with ringed sideroblasts**	1(2)	0(0)
** Refractory anemia with excess blasts**	20(43)	15(75)
** Refractory anemia with excess blasts in transformation**	2(4)	2(10)
** Chronic myelomonocytic leukemia**	1(2)	1(5)
**IPSS risk category-no.(%)**		
** Low**	7(16)	1(5)
** Intermediate 1**	17(38)	3(15)
** Intermediate 2**	14(31)	10(50)
** High**	7(16)	6(30)
**Duration of disease-mo**		
** Median**	1	0
** Range**	0–94	0–28
**Karyotype-no.(%)**		
** Non-del(5q)**	37(80)	15(75)
** Del(5q)/Monosomy 5**	9(20)	5(25)

IPSS is not available for one patient.

aCGH, array comparative genomic hybridization.

### Array comparative genomic hybridization

To complement the sequence-based studies, we used array comparative genomic hybridization (CGH) to measure DNA copy number across chromosome 5 that might be missed by cytogenetics or resequencing. We detected chromosome 5 deletions using the array CGH platform in 4/5 samples that were known to have del(5q) using cytogenetics (**Figure 1**, and data not shown). Array CGH did not detect del(5q) in one MDS sample that had 1/20 metaphases with a del(5)(q22q34) (**Figure 1**). This is consistent with our analysis of AML samples in which array CGH rarely detects copy number changes if the neoplastic clone comprises <25% of bone marrow cells (judged by the percent abnormal metaphases) (Walter, et al., submitted). None of the del(5q) samples had detectable deletions on the remaining wild-type chromosome (**Figure 1**, and data not shown), and no cytogenetically silent microdeletions were detected in the non-del(5q) samples (data not shown).

### Nucleotide changes in 28 chromosome 5q31.2 genes

To define the limit of detection for this DNA resequencing platform, we screened 90 MDS samples for FLT3 internal tandem duplications (ITD) using standard agarose gel electrophoresis and the Agilent LabChip. We detected 3 ITD+ samples and chose one for further analysis. We performed 6 serial dilutions of the ITD+ MDS DNA with FLT3 wild-type genomic DNA and performed PCR amplification followed by DNA sequencing of the PCR products. We could detect the FLT3 ITD when 12% of the alleles harbored the mutation, or when ∼20–25% of cells contain a heterozygous mutation (data not shown).

We designed and validated primers on control DNA templates for 479 amplicons covering the coding region and proximal introns of all 28 genes in the 5q31.2 interval (see **Table S2** for primer sequences). We then produced 7.5 megabases of double-stranded sequence for these 28 genes in the 46 samples. To ensure comprehensive coverage of all 28 genes, we performed bidirectional sequencing of amplicons and systematically tracked whether each coding nucleotide in all 28 genes was covered once or twice in the 46 samples. On average, 94% of all coding bases were covered, and 88% of bases were sequenced on both strands (**Table 2**). Only 10 amplicons failed to produce adequate sequence coverage using our resequencing pipeline, and 7 of these yielded high quality sequence after primers were redesigned. One exon in NME5, CTNNA1, and LOC729429 failed for all 46 samples, accounting for their low percent coverage (**Table 2**).

**Table 2:** Coverage report, nonsynonymous and splice site nucleotide changes in de novo MDS

Gene	Reference sequence	Coding (#bases/patient)	Total coverage(%) (Double/Single)	Identified Novel SNPs	Identified db SNPs
SPOCK1	NM_004598.3	1,320	95.2 (80.2/15.0)	0	0
KLHL3	NM_017415.1	1,764	96.0 (83.0/13.0)	0	0
HNRPA0	NM_006805.3	918	99.6 (95.3/4.3)	0	0
NPY6R*	NR_002713.1#	1953‡	100.0 (97.7/2.3)	0	2
MYOT	NM_006790.1	1,497	99.9 (98.3/1.6)	1	1
PKD2L2	NM_014386.1	1,829	99.9 (94.7/5.2)	2^II^	2
C5orf5	NM_016603.1	2,748	98.1 (90.2/7.9)	0	1
WNT8A	NM_058244.2	1,056	95.7 (87.4/8.3)	0	0
NME5	NM_003551.2	639	75.5 (58.5/17.0)	0	1¶
BRD8	NM_139199.1	3,708	96.0 (86.1/9.9)	2	1
KIF20A	NM_005733.1	2,673	98.3 (93.1/5.2)	2	0
CDC23	NM_004661	1,778	99.7 (95.4/4.3)	0	0
GFRA3	NM_001496.3	1,203	99.1 (90.3/8.8)	0	0
CDC25C	NM_001790.3	1,421	96.4 (90.7/5.7)	0	2
FAM53C	NM_016605.1	1,179	99.4 (92.4/7.0)	0	0
JMJD1B	NM_016604.3	5,286	94.8 (84/10.8)	1	2
REEP2	NM_016606.2	759	99.0 (90.9/8.1)	0	0
EGR1	NM_001964.2	1,632	99.5 (90.5/9.0)	0	0
ETF1	NM_004730.1	1,314	97.2 (90.7/6.5)	0	0
HSPA9	NM_004134	2,041	98.3 (88.5/9.8)	0	0
SNORD63+	NR_002913.1#	68‡	100.0 (99.2/0.8)	0	0
LOC391836+ *	XR_016942.1#	1063§	99.9 (98.0/1.9)	2	5
LOC729429	XM_001130232.1#	645	13.0 (13.0/0.0)	0	0
CTNNA1	NM_001903.2	2,721	94.1 (86.5/7.6)	0	1
LRRTM2	NM_015564.1	1,551	100.0 (99.0/1.0)	0	0
SIL1	NM_022464.4	1,386	100.0 (97.4/2.6)	1	0
MATR3	NM_018834.4	2,544	97.5 (94.3/3.2)	0	0
SNORA74A+	NR_002915.1#	200‡	99.0 (94.1/4.9)	1	0
Total		46,896		12	18

* Pseudogene.

+ Small RNA.

# Sequence was assembled using NT_034772.

‡ Non-coding gene, the number of bases/patient refers to the exon region.

§ Non-coding gene, the number of bases/patient refers to the whole gene.

II Reference sequence is NM_014386.2.

¶ Spice site SNP.

A semiautomated analysis pipeline was used to identify sequence variants [14]. We restricted our analysis to nonsynonymous and splice site nucleotide changes. Variants not found in dbSNP (build 129) were resequenced in bone marrow and paired germline (skin) samples. No somatic nonsynonymous or splice site mutations were detected in the 28 genes (**Table 2**). However, 12 novel SNPs were identified (**Table 3**). Nine of these were nonsynonymous, including a stop codon in *PKD2L2*, and 3 occurred in non-coding genes. The minor allele frequency for these 12 SNPs was 1.1–4.7% in this MDS population.

**Table 3:** Novel SNPs in MDS samples

Gene	Position	Variant	Codon	n‡	AA	AB	BB	MAF
BRD8	137514539	T>A	C972S	43	38(0.90)	4(0.10)	0(0.00)	0.047
BRD8	137526901	G>T	G635V	46	44(0.98)	1(0.02)	0(0.00)	0.011
JMJD1B	137736397	A>T	I110F	44	42(0.98)	1(0.02)	0(0.00)	0.011
KIF20A	137543399	C>T	S44F	45	42(0.95)	2(0.05)	0(0.00)	0.022
KIF20A	137548492	A>G	N594S	46	44(0.98)	1(0.02)	0(0.00)	0.011
LOC391836	137974309	G>A	NA	46	43(0.96)	2(0.04)	0(0.00)	0.022
LOC391836	137982403	A>G	NA	46	44(0.98)	1(0.02)	0(0.00)	0.011
MYOT	137239505	G>C	E149Q	46	44(0.98)	1(0.02)	0(0.00)	0.011
PKD2L2	137299391	C>T	Q560STOP	46	44(0.98)	1(0.02)	0(0.00)	0.011
PKD2L2	137303728	G>A	R612Q*	46	44(0.98)	1(0.02)	0(0.00)	0.011
SIL1	138406293	C>T	T123I	45	42(0.95)	2(0.05)	0(0.00)	0.022
SNORA74A	138642542	T>C	NA	46	44(0.98)	1(0.02)	0(0.00)	0.011

* Reference sequence is NM_014386.2.

‡ Patient 176267 had del(5)(q13q33) in 20/20 metaphases and complete loss of heterozygosity at all SNPs, which only contributed one allele to the calculation of allele frequency, and was excluded from the calculation of genotype frequency.

LOC391836 and SNORA74A are non-coding genes.

NA indicates not available; A, major allele; B, minor allele; and MAF, minor allele frequency.

### SNP frequencies of the 28 chromosome 5q31.2 genes in MDS vs. race-matched controls

To assess whether germline alleles of chromosome 5q31.2 genes are associated with MDS, we compared both the allele and genotype frequencies of all known SNPs in these genes (23 nonsynonymous, 19 synonymous, and 5 from non-coding genes) in the 45 MDS patients of European ancestry from our cohort to race-matched HapMap controls. The minor allele frequency for 16/47 SNPs was less than 5% in all MDS or race-matched control samples, leaving 31 SNPs that could be analyzed with this sample size (**Table S3**). The minor allele frequencies of two coding SNPs were significantly over-represented in MDS cases vs. race-matched controls: rs3734166 (*CDC25C* R70C) (OR 1.836, 95% conf 1.030–3.272, p = 0.048), and rs2905608 (*KLHL3* A157A) (OR 99.47, 95% conf 5.914–1673, p = 2.187E-10). We used pyrosequencing to genotype 190 additional samples from normal individuals of European descent (cancer free controls from Washington University, and HD100 controls) for SNPs rs3734166 and rs2905608. There was no significant difference in allele or genotype frequencies for rs3734166 (p = 0.246, p = 0.450, respectively) and rs2905608 (p = 0.214, p = 0.182, respectively) after pooling all available control data (**Table 4**). This study was powered to detect changes in minor allele frequencies that occurred in at least 10% of MDS cases, therefore, race-matched control samples were not genotyped for the 12 novel SNPs identified in our 46 MDS samples because they occurred at such low frequencies (1.4–4.7%).

**Table 4:** Allele and genotype frequencies for CDC25C(R70C) and KLHL3(A157A) SNPs in MDS and race-matched control samples

Gene	db SNP	Position	Variant	Codon	Category	MDS	All Control	HD100 coriell control	CFC control	HapMap control
CDC25C	rs3734166	137693222	C>T	R70C	**n** *	45	271	87	94	90
					**AA**	20(0.45)	147(0.54)	42(0.48)	49(0.52)	56(0.62)
					**AB**	20(0.45)	109(0.40)	37(0.43)	40(0.43)	32(0.36)
					**BB**	4(0.09)	15(0.06)	8(0.09)	5(0.05)	2(0.02)
					**MAF**	0.315	0.256	0.305	0.266	0.200
					**P Value, MDS vs Control (Allele** * **/Genotype** * **)**		0.246/0.450	0.888/0.948	0.475/0.613	**0.048**/0.070
KLHL3	rs2905608	137055928	G>A	A157A	**n** *	42	271	94	95	82
					**AA**	24(0.59)	194(0.72)	58(0.62)	54(0.57)	82(1.00)
					**AB**	15(0.37)	63(0.23)	27(0.29)	36(0.38)	0(0.00)
					**BB**	2(0.05)	14(0.05)	9(0.10)	5(0.05)	0(0.00)
					**MAF**	0.229	0.235	0.239	0.242	0.000
					**P Value, MDS vs Control (Allele** * **/Genotype** * **)**		0.214/0.182	1.000/0.500	0.878/0.982	**2.187E-10/1.000E-04**

* Patient 176267 had del(5)(q13q33)[20/20] and complete loss of heterozygosity at all SNPs,which only contributed one allele to the calculation of allele frequency, and was excluded from the calculation of genotype frequency.

Bold indicates a siginificant difference at P<0.05.

CFC indicates cancer free control. Other abbreviations are explained in Table 3.

### SNP loss of heterozygosity in del(5q) samples

**Figure 2:**
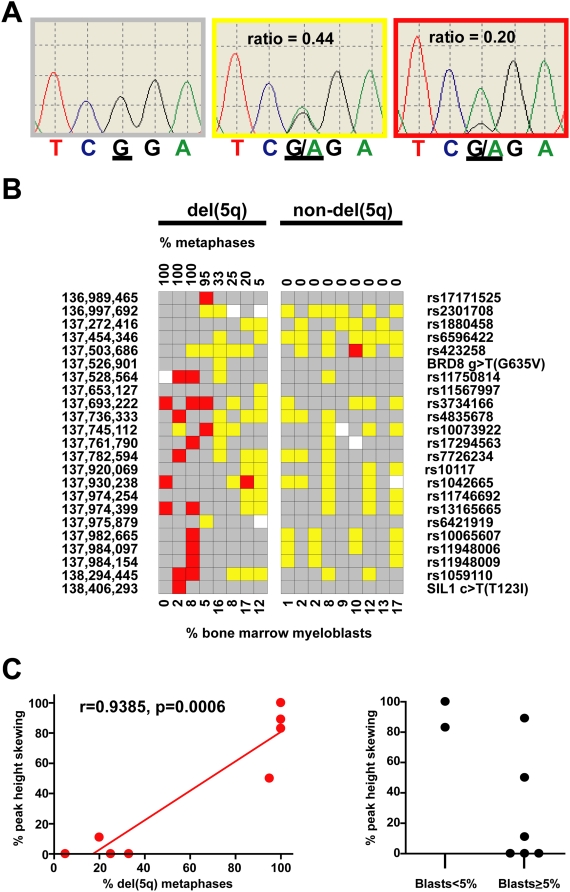
5q31.2 SNP loss of heterozygosity in del(5q) samples. A. Sequence traces for SNP rs1059110 in tumor samples from an individual homozygous for the common allele (left), a heterozygote (middle) with a normal allele trace peak height ratio = 0.44 (i.e., no allelic imbalance), and a heterozygote (right) with an abnormal allele trace peak height ratio = 0.20 (i.e., allelic imbalance). B. Heat map of allelic imbalance for 23 SNPs from 8 del(5q) and 9 non-del(5q) MDS samples. Each column represents a sample, and each row represents a SNP. red, allelic imbalance; yellow, no evidence of allelic imbalance; grey, homozygous SNP; white, no data. Base pair location is listed in the left column. C. (left panel) Proportion of abnormal metaphases is highly correlated with allelic skewing in heterozygous SNPs. (right panel) Blast count is a poor predictor of clonality. Two samples with bone marrow myeloblast counts less than 5% are clonal, indicated by marked SNP allelic skewing. In contrast, four samples with myeloblast counts greater than or equal to 5% were non-clonal as measured by SNP allelic ratio.

To assess whether preferential retention of a SNP allele occurred in the 9 monosomy 5 or del(5q) samples, we examined their sequence traces for 64 SNPs (12 SNPs in **Table 3**, 47 SNPs in **Table S3**, and 5 SNPs without race-matched control data). DNA extracted from a homogeneous population of tumor cells harboring a deletion of one copy of chromosome 5q31.2 will contain complete loss of heterozygosity for all SNPs in 5q31.2 genes that are heterozygous in the germline. Sequence traces from these bone marrow samples display only one peak at each SNP, which represents the retained SNP allele in the tumor cells (**Figure 2**). In contrast, DNA extracted from a heterogeneous population of cells containing both tumor cells with a del(5q), and normal cells without del(5q), produce sequence traces displaying two alleles of unequal peak heights indicating allele skewing. There were 5 SNPs that had allele skewing in 3 or more del(5q) samples, but 0/5 SNPs displayed uniform retention of the same allele (data not shown).

To address the possibility that the cellular heterogeneity present in MDS bone marrow samples could limit the use of unfractionated cells for DNA based genomic studies, we used the SNP resequencing data to determine whether we could detect clonality in MDS bone marrow samples that had less than 5 percent myeloblasts. We calculated the allele trace peak height ratio (peak height of allele A/(peak heights of alleles A+B)) for biallelic SNPs in tumor DNA samples (bone marrow). The allele peak height ratio was considered abnormal, indicating allelic imbalance, if the ratio was ≥0.7 or ≤0.3 (**Figure 2**). The del(5q) samples had bone marrow myeloblast counts ranging from 0–17%, and an abnormal chromosome 5q31.2 in 5–100% of their bone marrow cell metaphases. The SNP allelic frequencies were highly correlated with the percent of abnormal metaphases in 8 del(5q) samples (r = 0.9385, p = 0.0006), indicating that the sequencing data accurately reflects clonality (**Figure 2**). In contrast, the bone marrow myeloblast count is a poor surrogate for clonality. Even at blast counts less than 5 percent, clonal cells are detected using the SNP peak height skewing data, implying that the majority of bone marrow cells may be clonal even when samples have low bone marrow myeloblast counts (**Figure 2**).

### Gene expression levels

**Figure 3:**
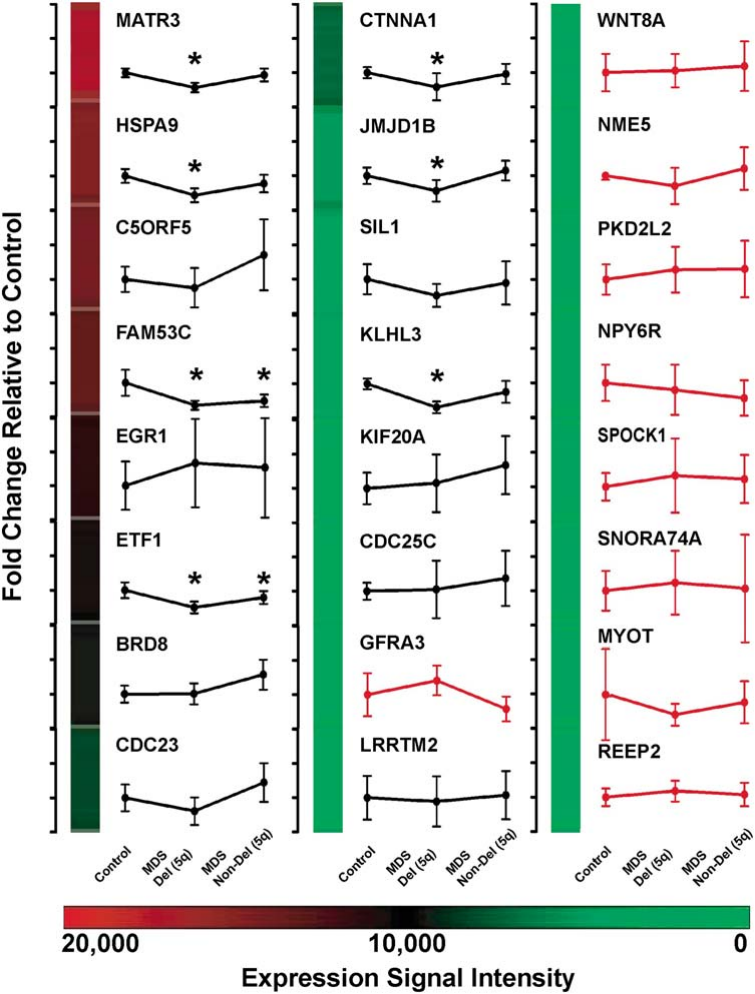
Gene expression levels of del(5q) genes in CD34+ cells. mRNA expression levels were determined for 24 del(5q) genes that had probesets on the Affymetrix U133plus2 array using total RNA extracted from CD34+ purified cells from 6 MDS patients with del(5q), 20 MDS patients without del(5q), and 6 normal control individuals. Genes were rank ordered based on the median signal intensity in control samples. The average signal intensity of MDS samples (± standard deviation) with or without del(5q) is plotted relative to control samples. Each tick mark on the y-axis represents 1 fold change relative to control samples. The color bar indicates the Affymetrix signal intensity values for the control samples for each gene. Genes with 70–100% of samples called absent are plotted with red lines. When multiple probesets were called present for a gene, probesets were averaged (probesets were included in the average signal if they were present in at least 4 of 6 control samples and had a median expression in controls greater than 1,500, the scaled target intensity of arrays). If all probesets were called absent for a gene, the highest median expressing probeset was used for plotting. * p<0.05 compared to control.

To assess whether del(5q) gene expression may be affected by mechanisms other than coding or splice site DNA mutations (i.e., mutations in non-coding DNA or epigenetic alterations), we compared the mRNA levels of 28 del(5q) genes in CD34+ purified cells from 6 MDS patients with del(5q), 20 MDS patients without del(5q), and 6 normal donors using the Affymetrix U133plus2 array. Four of the 28 genes did not have a probeset on the array (*HNRPA0, LOC391836, LOC729429, SNORD63*). The remaining 24 genes were rank ordered based on their median mRNA expression level (Affymetrix units) in the 6 control samples, and the average expression level of a gene for MDS patients with del(5q) (n = 6) and MDS patients without del(5q) (n = 20) was plotted relative to the level in control samples (**Table S4**, **Figure 3**). Nine of the 24 genes were called absent in 70–100% of all 32 samples (colored red in **Figure 3**). Consistent with haploinsufficiency, 7 genes had a significant reduction in average signal intensity in MDS samples with del(5q) compared to control samples (0.47 average fold change for MATR3, HSPA9, FAM53C, ETF1, CTNNA1, JMJD1B, and KLHL3 relative to control samples, p<0.04) and compared to MDS samples without del(5q) (0.52 average fold change relative to non-del(5q) samples, p<0.02 excluding FAM53C). No gene had mRNA levels in del(5q) samples consistently below the expected haploinsufficient level of 50%, indicating that biallelic loss of expression is not common in CD34+ samples containing del(5q). However, FAM53C and ETF1 were expressed at significantly lower levels in MDS samples without del(5q) compared to control samples (0.47 and 0.79 fold change relative to control, respectively, p≤0.03), suggesting that these genes might be altered in the absence of a cytogenetic deletion. To verify this possibility, we performed quantitative RT-PCR for FAM53C using available total RNA from CD34+ cells. Quantitative RT-PCR did not confirm the Affymetrix array results for haploinsufficiency of FAM53C (data not shown). Quantitative RT-PCR was not performed for ETF1 because only 1 of 2 probesets on the array showed reduced expression in non-del(5q) samples. In summary, of the 15 chromosome 5q31.2 genes with detectable expression by microarray, none was consistently expressed out of proportion to gene dosage.

## Discussion

In this study, we determined by high-throughput resequencing and array comparative genomic hybridization (CGH) that point mutations and small deletions in chromosome 5q31.2 genes are not common events in *de novo* MDS. A critical aspect of this study design is that the nucleotide sequence and copy number for all of the genes in the commonly deleted segment were examined comprehensively in a large number of MDS patients without del(5q). These results therefore demonstrate that a cytogenetic deletion spanning the commonly deleted segment on chromosome 5q31.2 is the predominant genetic event leading to haploinsufficiency of multiple del(5q) genes. These results also imply that the simultaneous loss of multiple genes may be necessary for disease initiation and progression of del(5)(q31.2) associated MDS and AML.

Haploinsufficiency of three 5q31.2 candidate genes (*HSPA9, CTNNA1, EGR1*) may contribute to disease pathogenesis, but current data suggests that these genes individually do not recapitulate all features of MDS [8], [16], [17]. Zebrafish carrying an ENU-induced heterozygous mutation in the ortholog of the human *HSPA9* gene display increased apoptosis in blood cells characteristic of ineffective hematopoiesis [16]. Look and colleagues found that decreased CTNNA1 expression in CD34+CD38-CD123+Lin- cells from high risk del(5q) associated MDS or AML patients was associated with methylation of the *CTNNA1* promoter, but not with point mutations of the residual allele in primary tumor cells [8]. However, others found no reduction in CTNNA1 mRNA levels below 50% in CD34+CD38-Thy1+ cells from low risk del(5q) MDS patients [18]. Most recently, Le Beau and colleagues observed that *Egr1* heterozygous mutant mice have normal resting hematopoiesis, but develop myeloid diseases after treatment with ENU [17]. Collectively, the data implicate that more than one gene is likely to be involved in the pathogenesis of MDS, and support the hypothesis that haploinsufficiency of multiple 5q31.2 genes contributes to MDS initiation or progression.

It is unlikely that a recurrent, clonally dominant, genetic event (mutation, gene silencing, or polymorphism) in these 28 genes was not detected in this *de novo* MDS cohort due to cellular heterogeneity or sample size. Although cellular heterogeneity in MDS bone marrow samples exists, our resequencing (**Figure 2**) and array CGH (**Figure 1**) results confirm findings from previous studies using interphase fluorescent in situ hybridization (FISH) for del(5q), which concluded that the myeloblast count often underestimates the proportion of cells that are clonally derived in MDS [19], [20], [21]. In addition, by sequencing 37 MDS samples without del(5q), there is a 0.9543 probability of detecting a 5q31.2 gene mutation if it occurs in at least 8% of MDS samples without a cytogenetic del(5q). Furthermore, we found no evidence of biallelic loss of mRNA expression of del(5q) genes in CD34+ cells harvested from our MDS patients with del(5q), and we found no SNP significantly associated with MDS or evidence of preferential retention of a residual SNP allele in MDS samples with del(5q).

Collectively, our data provides direct evidence supporting the hypothesis that haploinsufficiency of multiple genes in the commonly deleted segment is the most likely relevant genetic consequence of deletions spanning chromosome 5q31.2. Moving forward and testing the hypothesis of haploinsufficiency as a disease mechanism is difficult, and using mouse models is problematic. The syntenic chromosome 5q31.2 region in mouse resides on two chromosomes, making it difficult to accurately engineer a complete knockout of all the genes at once. In addition, creating individual targeted knockout mice for each candidate gene is time-consuming, cost-prohibitive, and impractical. However, it is now possible to perform comprehensive high-throughput gene knockdown screens using RNAi technology to model haploinsufficiency because lentiviral short hairpin libraries exist [22], [23]. This approach has yielded important information for the *RPS14* gene located in the chromosome 5q33.1 commonly deleted segment in patients with the 5q minus syndrome [24], and this technology will expand our understanding of single gene and gene combinations that may contribute to disease initiation in MDS and AML. Ultimately, identifying the genetics events leading to MDS initiation may provide insight into targeted therapy for MDS and AML.

## Supporting Information

Table S1 (0.06 MB XLS)

Table S2 (0.13 MB XLS)

Table S3 (0.14 MB XLS)

Table S4 (0.09 MB XLS)
